# Supplementary material for: Causal Relationship between Aging and Anorexia Nervosa: A White-Matter-Microstructure-Mediated Mendelian Randomization Analysis
Source: Biomedicines. 2024 Aug 16;12(8):1874. doi: 10.3390/biomedicines12081874 (PMC11351342; doi:10.3390/biomedicines12081874)
Supplement: Supplementary file 1 [file biomedicines-12-01874-s001.zip › Supplementary Figure/Figure S4/Singe SNP plot of MR analysis for TL to ubm-b-2065.pdf]

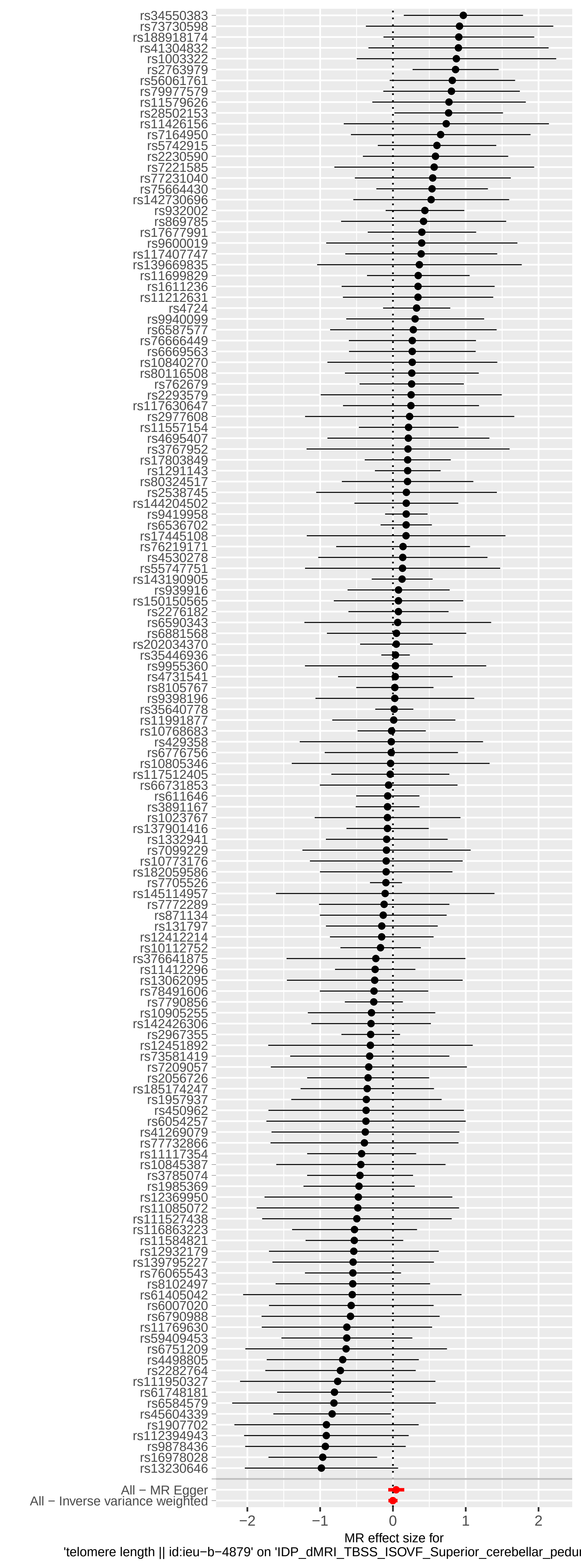
rs34550383
rs73730598
rs188918174
rs41304832
rs1003322
rs2763979
rs56061761
rs79977579
rs11579626
rs28502153
rs11426156
rs7164950
rs5742915
rs2230590
rs7221585
rs77231040
rs75664430
rs142730696
rs932002
rs869785
rs17677991
rs9600019
rs117407747
rs139669835
rs11699829
rs1611236
rs11212631
rs4724
rs9940099
rs6587577
rs76666449
rs6669563
rs10840270
rs80116508
rs762679
rs2293579
rs117630647
rs2977608
rs11557154
rs4695407
rs3767952
rs17803849
rs1291143
rs80324517
rs2538745
rs144204502
rs9419958
rs6536702
rs17445108
rs76219171
rs4530278
rs55747751
rs143190905
rs939916
rs150150565
rs2276182
rs6590343
rs6881568
rs202034370
rs35446936
rs9955360
rs4731541
rs8105767
rs9398196
rs35640778
rs11991877
rs10768683
rs429358
rs6776756
rs10805346
rs117512405
rs66731853
rs611646
rs3891167
rs1023767
rs137901416
rs1332941
rs7099229
rs10773176
rs182059586
rs7705526
rs145114957
rs7772289
rs871134
rs131797
rs12412214
rs10112752
rs376641875
rs11412296
rs13062095
rs78491606
rs7790856
rs10905255
rs142426306
rs2967355
rs12451892
rs73581419
rs7209057
rs2056726
rs185174247
rs1957937
rs450962
rs6054257
rs41269079
rs77732866
rs11117354
rs10845387
rs3785074
rs1985369
rs12369950
rs11085072
rs111527438
rs116863223
rs11584821
rs12932179
rs139795227
rs76065543
rs8102497
rs61405042
rs6007020
rs6790988
rs11769630
rs59409453
rs6751209
rs4498805
rs2282764
rs111950327
rs61748181
rs6584579
rs45604339
rs1907702
rs112394943
rs9878436
rs16978028
rs13230646
All − MR Egger
All − Inverse variance weighted
−2
−1
0
1
2
MR effect size for
'telomere length || id:ieu−b−4879' on 'IDP_dMRI_TBSS_ISOVF_Superior_cerebellar_pedu
